# Digital Health Solutions to Reduce the Burden of Atherosclerotic Cardiovascular Disease Proposed by the CARRIER Consortium

**DOI:** 10.2196/37437

**Published:** 2022-10-17

**Authors:** Bart Scheenstra, Anke Bruninx, Florian van Daalen, Nina Stahl, Elizabeth Latuapon, Maike Imkamp, Lianne Ippel, Sulaika Duijsings-Mahangi, Djura Smits, David Townend, Inigo Bermejo, Andre Dekker, Laura Hochstenbach, Marieke Spreeuwenberg, Jos Maessen, Arnoud van 't Hof, Bas Kietselaer

**Affiliations:** 1 Department of Cardiothoracic Surgery Maastricht University Medical Center+ Maastricht Netherlands; 2 Cardiovascular Research Institute Maastricht Maastricht Netherlands; 3 Department of Radiation Oncology (MAASTRO) GROW School for Oncology and Developmental Biology Maastricht university Medical Center+ Maastricht Netherlands; 4 Department of Health, Ethics and Society Faculty of Health, Medicine and Life Sciences Maastricht University Maastricht Netherlands; 5 Department of Health Services Research Faculty of Health, Medicine and Life Sciences Maastricht University Maastricht Netherlands; 6 Department of Data Science and Knowledge Engeneering Maastricht University Maastricht Netherlands; 7 Statistics Netherlands Heerlen Netherlands; 8 The Netherlands eScience Center Amsterdam Netherlands; 9 Care and Public Health Research Institute Faculty of Health, Medicine and Life Sciences Maastricht University Maastricht Netherlands; 10 Department of Health Services Research Maastricht University Maastricht Netherlands; 11 Department of Cardiology Zuyderland Medical Centre Heerlen Netherlands; 12 Department of Cardiology Maastricht University Medical Center+ Maastricht Netherlands

**Keywords:** atherosclerotic cardiovascular disease, ASCVD, cardiovascular risk management, CVRM, eHealth, digital Health, personalized e-coach, big data, clinical prediction models, federated data infrastructure

## Abstract

Digital health is a promising tool to support people with an elevated risk for atherosclerotic cardiovascular disease (ASCVD) and patients with an established disease to improve cardiovascular outcomes. Many digital health initiatives have been developed and employed. However, barriers to their large-scale implementation have remained. This paper focuses on these barriers and presents solutions as proposed by the Dutch CARRIER (ie, Coronary ARtery disease: Risk estimations and Interventions for prevention and EaRly detection) consortium. We will focus in 4 sections on the following: (1) the development process of an eHealth solution that will include design thinking and cocreation with relevant stakeholders; (2) the modeling approach for two clinical prediction models (CPMs) to identify people at risk of developing ASCVD and to guide interventions; (3) description of a federated data infrastructure to train the CPMs and to provide the eHealth solution with relevant data; and (4) discussion of an ethical and legal framework for responsible data handling in health care. The Dutch CARRIER consortium consists of a collaboration between experts in the fields of eHealth development, ASCVD, public health, big data, as well as ethics and law. The consortium focuses on reducing the burden of ASCVD. We believe the future of health care is data driven and supported by digital health. Therefore, we hope that our research will not only facilitate CARRIER consortium but may also facilitate other future health care initiatives.

## Introduction

Atherosclerotic cardiovascular disease (ASCVD) remains one of the leading causes of death worldwide [1] and is a burden to medical expenses in Europe [2]. The occurrence of ASCVD is highly correlated with conventional risk factors such as high blood pressure and smoking. Therefore, prevention and treatment of risk factors is of importance in reducing this burden. ASCVD can be prevented to a great extent by a healthy lifestyle [3].

However, a recent survey from EUROASPIRE V investigators showed that “a large majority of patients at high [AS]CVD risk fail to achieve lifestyle, blood pressure, lipid, and glycemic targets” [4]. The limited adherence to these targets is one of the causes of the remaining burden of ASCVD. Therefore, there is an unmet clinical need for innovative solutions to support people at risk, patients with an established disease, and their health care professionals to improve cardiovascular outcomes.

eHealth has the potential to reach a wide audience of people at risk and support patients to adopt a healthy lifestyle and reduce their cardiovascular risk [5-7]. Nevertheless, there are barriers to large-scale implementation of digital health as stated by the Society of Cardiology e-Cardiology working group in their recent position paper [8].

One of the barriers to eHealth implementation is a mismatch between the end product and the needs of its end users [8-10]. It is known that specific groups have a low adherence to eHealth (eg, people in older age and those with low health literacy). Involvement of relevant stakeholders (eg, physicians and patients) during development (or so-called cocreation) enhances the adoption of the end product by its end users [11].

In addition to cocreation with relevant stakeholders, implementation also depends on availability and communicability of data between stakeholders involved in the prevention of ASCVD. The availability of data is essential for innovative clinical prediction models (CPMs) for the early identification of people at risk, accurate risk estimation, and guiding interventions [12].

Unfortunately, data are scattered among the stakeholders, and thus, not readily available. Therefore, a mature data infrastructure connecting the stakeholders needs to be developed. A federated data infrastructure using the Personal health Train [13] is a promising technology to connect stakeholders.

Finally, successful implementation of digital health interventions requires consideration of ethical and legal demands for the aforementioned data infrastructure. Therefore, an ethical and legal framework for responsible data handling in health care needs to be instigated.

The Dutch CARRIER (ie, Coronary ARtery disease: Risk estimations and Interventions for prevention and EaRly detection) consortium (Table 1) consists of a collaboration between experts in the fields of eHealth development, ASCVD, public health, big data, and ethics and law. The consortium was established in 2020 and is funded by the Dutch Research Council. CARRIER targets early identification, prevention, and treatment of ASCVD, with a regional alliance in the south of the Netherlands. We believe the future of health care is data driven and supported by digital health. Therefore, we collaborate on research of big data-driven, participative self-care interventions to reduce the burden of ASCVD.

In this paper, we will discuss the aforementioned barriers and propose the following practical solutions for the implementation of digital health to reduce the burden of ASCVD: (1) description of the development process of an eHealth application using cocreation with relevant stakeholders, which enhances the adoption of the end product by its end users; (2) presentation of the modeling approach for two innovative CPMs for the early identification of people at risk of developing ASCVD, accurate risk estimation, and guiding interventions; (3) description of a federated data infrastructure using the Personal Health Train to connect relevant stakeholders; and (4) discussion of an ethical and legal framework for responsible data handling in health care.

**Table 1:** Terms and abbreviations used within CARRIER consortium and their definitions.

Term or abbreviation	Definition
Digital health	A broad umbrella term encompassing the whole technical solution CARRIER proposes
eHealth	The product that is used by the end users
ASCVD	Atherosclerotic cardiovascular disease
Design thinking	A cyclic process to develop products
Cocreation	Involvement of relevant stakeholders during the development of products
CPM	Clinical prediction model
Implementation	The process that ensures the end product is used in daily practice
Federated learning	A technique that trains algorithms using data from decentralized organizations
Federated data infrastructure	A set of tools and processes that allows federated learning
Ethical and legal framework	The framework that needs to be created to ensure responsible data handling
SN	Statistics Netherlands—the national office for statistics
EHR	Electronic health record
NLP	Natural language processing, or text mining—a technique to analyze human language
FAIR data	Findable, Accessible, Interoperable, and Reusable data
Vertically partitioned	Data from the same individual that is distributed among different organizations
Record linkage	Linking records that correspond to the same individual across different data sets
SMC	Secure multiparty computation, which allows organizations to perform calculations with private data without revealing the data
ε-Differential privacy	The degree of privacy sensitivity of an analysis
GDPR	General Data Protection Regulation

## The Development Process of a Digital Health Solution

**Figure 1:**
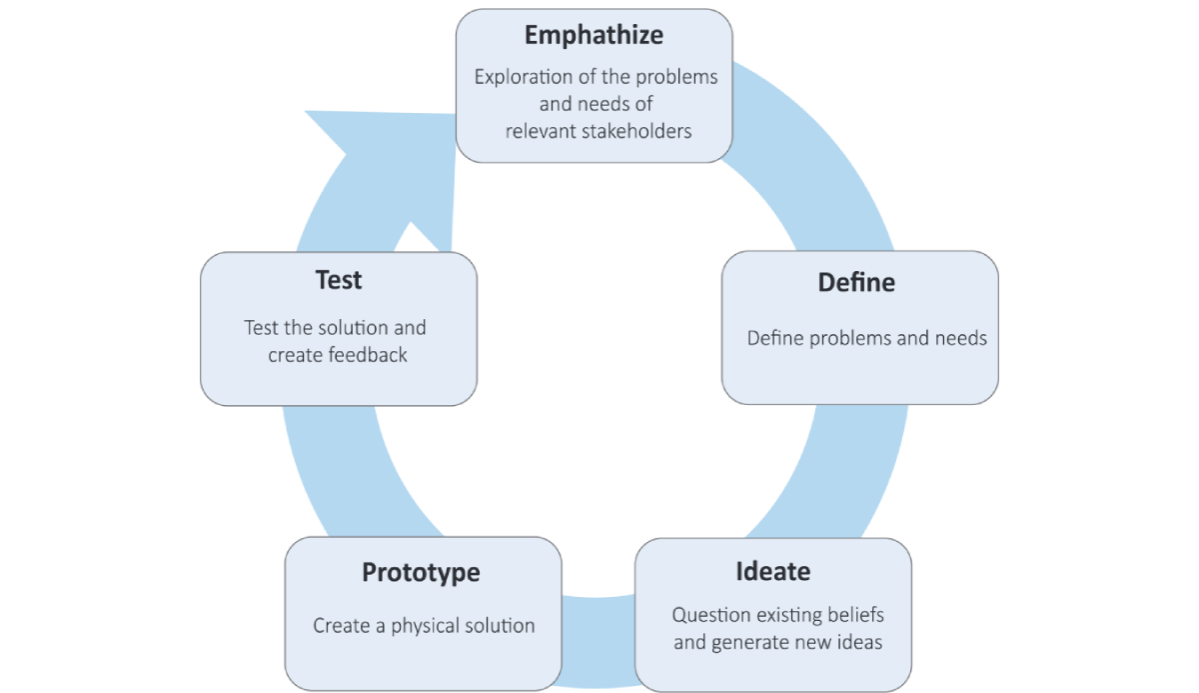
The 5 iterative phases of design thinking.

To increase the likelihood of successful adoption of the end product by its end users, the user-centered design approach—“design thinking”—will be applied [14,15]. Design thinking is an iterative process to create and evaluate innovative solutions; this means that end users and relevant stakeholders will be involved throughout the development process to ensure the final product meets the needs and vision of all relevant parties. Design thinking consists of 5 iterative phases (Figure 1).

**Figure 2:**
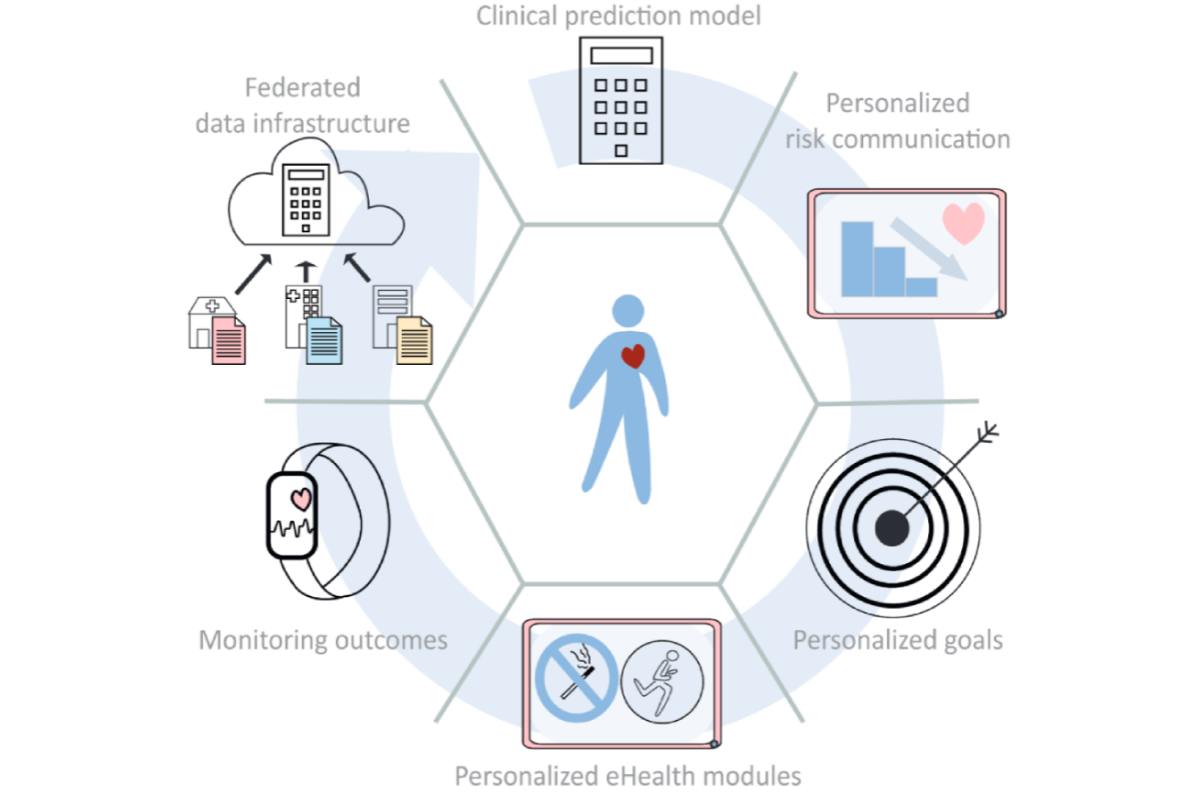
Proposed digital health solution.

Within the first 2 phases (ie, empathize and define), we used cocreation to describe our ideal digital health solution for ASCVD. Essential elements of this solution are as follows: a CPM for risk estimation, personalized risk communication, personalized treatment goals, individualized eHealth modules to guide and monitor treatment and outcomes, and the use of a federated data infrastructure to connect different organizations involved (Figure 2). These elements are explained in more detail in the next sections.

Integration of CPMs in a digital health solution enables the use of relevant and readily available data from different organizations to identify people at risk. After calculating the risk, this will be visualized and communicated according to the preferences and level of understanding of the individual. This information supports the patient and health care professional to set tailored goals using shared decision-making. The optimal format and visualization for risk communication is further examined and is therefore part of the objectives of CARRIER.

As different subgroups of people have a different adherence to lifestyle interventions, eHealth modules need to be adapted toward a personalized approach [16]. When interventions are personalized, the content becomes more relevant to the individual. This will result in an increased adherence to the intervention and a greater improvement in health outcomes [17].

eHealth modules that support patients in achieving their goals will be available within the eHealth solution for cardiovascular risk factors, such as smoking, hypertension, diabetes, and hypercholesterolemia, and additionally, for medication adherence, a healthy diet, physical activity, and stress reduction. The modules should also provide the possibility to monitor the effect of the interventions by (automatic) collection of relevant data. Activity trackers, for example, can be used to obtain insight into the physical activity of the patient. The modules should provide personalized feedback to the patients and their health care professionals. Monitoring of the goals can assist the patient and health care professional to guide the timing and frequency of medical follow-up. Relevant outcomes should be collected and used to improve the content of the eHealth modules and the risk estimation of the CPMs. Besides the first 2 steps (ie, empathize and define) of the design thinking process, the other 3 steps include the following: ideate, prototype, and testing. The testing phase will include an evaluation with the involvement of end users. Thereby, the testing phase provides feedback and may lead to reevaluation of the proposed solution. To ensure maximal implementation, the eHealth solution needs to be integrated into the workflow of care pathways [8].

## The Modeling Approach for a Screening and Interventional Model

The early identification of people at risk for ASCVD can be supported by CPMs. However, current CPMs, such as Framingham or SCORE, only use conventional patient characteristics for their risk estimation [18]. To improve the accuracy of CPMs, nonconventional risk factors, such as ethnicity, socioeconomic status, obesity, and physical inactivity, can be added [12,19,20]. Adding nonconventional risk factors can identify people at increased risk without the need for prior medical testing (unlike measurement of high blood pressure and hypercholesterolemia) and can inform lifestyle interventions (eg, losing weight and increasing physical activity).

Two CPMs will be constructed in CARRIER. The first model aims to identify people at risk to develop ASCVD. The second model aims to guide interventions for those at a high risk or with an established ASCVD. We will refer to the first model as the screening model and to the second model as the intervention model.

Consensus on which conventional and nonconventional risk factors should be included in the models will be reached through a Delphi study [21] by experts in the field of ASCVD. The relationships among these factors and ASCVD will be depicted using a causal graph [22]. This is a graphical representation of the causal structure among the factors, in which arrows indicate the direction of the causal effects.

The screening model will be based on federated learning (explained in the next section) using data from three different organizations: hospitals, general practices, and the national office for statistics (in our case, Statistics Netherlands [SN]). Data from the hospitals and general practices include electronic health record (EHR) data from individual patients. EHR data are not systematically gathered, resulting in many possible sources of bias (eg, nonrandom missingness) [23], which we will adjust by applying a range of techniques [24]. Furthermore, EHR data sets contain much information in free-text form. We will explore to which extent natural language processing (or text mining) techniques aid to supplement the structured EHR data with data extracted from free text [25].

Regarding the intervention model, we plan to make use of data of a regional observational cohort study in which clinical and lifestyle data are being collected [26]. These data are required to construct a causal model that can estimate the effect of lifestyle interventions. The following two main strategies were identified to incorporate hypothetical interventions in CPMs [27]: combining a CPM with causal effects estimated in randomized controlled trials and estimating causal effects based on observational data alone. We will use both these strategies during the development of CPMs within CARRIER.

In terms of statistical modelling techniques, we plan to use (1) regression models; (2) Bayesian networks, which suit well to causal graphs and provide an intuitive and explainable framework where data can be combined with expert knowledge [28]; and (3) neural networks or deep learning, which have gained popularity in recent years due to model complex functions [29]. Established models such as SCORE2 [30] will be used to compare the models’ performance. After model development, we envision external validations in order to research the models’ transportability to different settings and related populations. Reporting of the development and validations of the CPMs will adhere to the Transparent Reporting of a Multivariable Prediction Model for Individual Prognosis or Diagnosis (TRIPOD) guidelines [31].

When implementing the screening and intervention model, we anticipate a deterioration of the models’ performance over time due to shifting data distributions [32]. Therefore, we aspire to schedule temporal validations and updates of the models. In addition, we aim to monitor predictors continuously to detect changes in their univariable distribution, which may trigger an earlier than planned check.

## The Federated Data Infrastructure to Support Our Digital Health Solution

The two main issues of using medical data for prediction modelling are poor quality and scattering across different organizations. CARRIER aims to tackle these problems by following the Findable, Accessible, Interoperable, and Reusable (FAIR) data principles [33] and by developing a federated data infrastructure. FAIR data principles establish a set of guidelines that lead to the improvement of data quality, such as the use of metadata and standard vocabularies and ontologies. The use of standards also allows for data from different organizations to be combined [34] and increases the chances of data being reusable for secondary purposes [35].

To develop our screening model, we need to combine data from different organizations. Currently, relevant and potentially privacy-sensitive data from different organizations need to be shared to a central database before they can be analyzed. However, growing awareness of privacy and data ownership–related ethical issues have led to growing legal restrictions on data sharing. This is noticeable under the current legal regimens (more details are provided in the next section).

**Figure 3:**
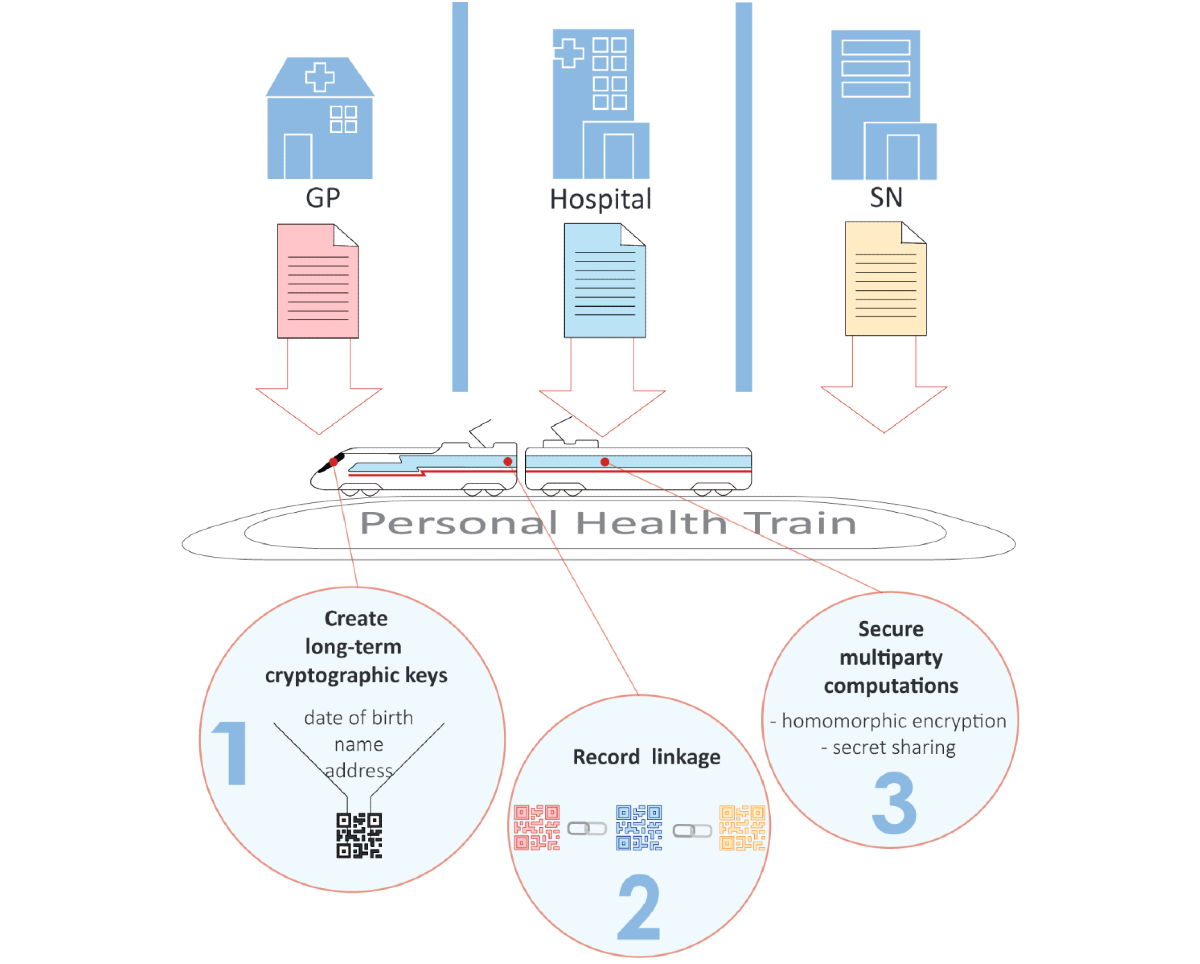
Federated data infrastructure. GP: general practices; SN: Statistics Netherlands.

In the last few years, federated learning has risen to prominence to analyze distributed data, for example, to train machine learning models [36,37]. Although the term federated learning seems to denote the algorithm, we will use the term ‘federated data infrastructure’ to denote the collection of tools and processes necessary to allow federated learning to work safely and reliably. As such, federated data infrastructures allow for decentralized data to be analyzed without subject-level data leaving the organization. This has implications on privacy preservation and on retaining control of data by its owner, which will allow for safer reuse of data [38]. In CARRIER, we will use the Personal Health Train [13], a federated learning platform that encompasses technical and legal aspects. This is implemented with an open-source software that handles the communication and authentication and provides an environment to implement federated algorithms [39]. The federated data infrastructure is visualized in Figure 3.

Data from hospitals, general practices, and SN will be used to develop the screening model for ASCVD. Hence, data on each individual are distributed across different organizations. This is termed ‘vertically partitioned’ (or ‘heterogeneous’). Federated learning on vertically partitioned data presents a unique set of challenges. The first is privacy-preserving record linkage, that is, linking records that correspond to the same individual across different data sets, without revealing any sensitive information [40]. Given that readily available identifiers, such as the citizens’ service number, are illegal to be used due to privacy concerns, we will use alternatives such as long-term cryptographic keys [41]. This technique uses personal characteristics, such as name, date of birth, and address, to create a unique encrypted code, which is used for record linkage.

**Figure 4:**
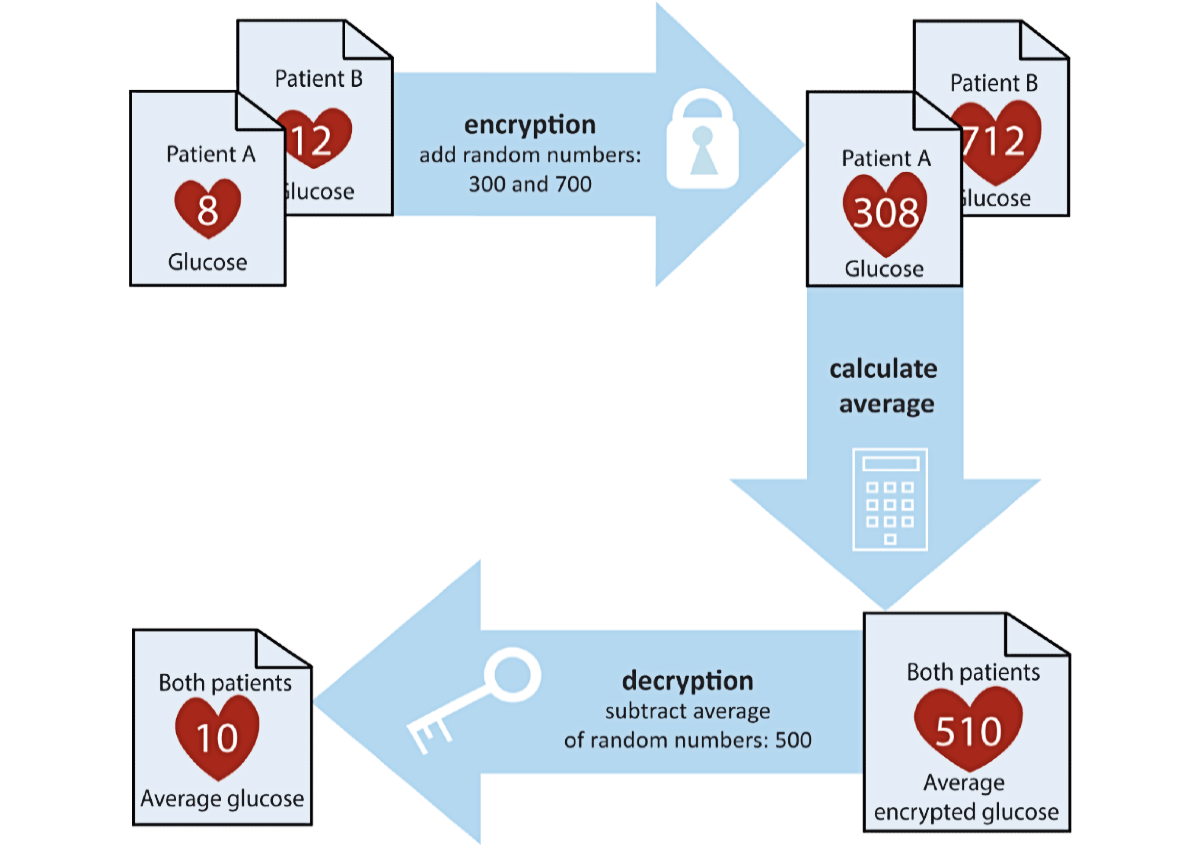
Homomorphic encryption of two fictive glucose levels.

After matching the individual records, the main challenge is to perform data analysis on different sources without each party revealing their data to the other parties. Secure multiparty computation (SMC) is a subfield of cryptography that allows a set of parties (or organizations) to perform calculations with their private data without revealing these data to the other parties [42]. A technique used in SMC is homomorphic encryption [43] (Figure 4), which is a form of encryption that allows computations on encrypted data. Another technique used in SMC is secret sharing [44], where numbers are ‘split’ across multiple organizations and can only be reconstructed after being combined. As such, SMC can be used to analyze data and even train models from different organizations without the data being revealed.

After requested analyses are performed within the federated data infrastructure, the degree of privacy sensitivity of an analysis can be represented by ε-differential privacy [45]. This can be used as a criterion before sharing an analysis from one source to the other. An analysis is ε-differentially private when its results do not change significantly with slight changes in the population. This guarantees that the results do not depend on the values of any given individual in the data set, and therefore, it is not possible to trace back information to an individual patient.

In summary, in CARRIER, we are developing a federated, FAIR data infrastructure. This infrastructure allows us to train models on vertically partitioned data and ensures that our analyses preserve privacy by means of SMC techniques and ε-differential privacy.

## The Development of an Ethical and Legal Framework for Responsible Data Handling in Health Care

CARRIER concerns the processing of different medical, lifestyle, and other personal data, held by different organizations, that relate to citizens. These data are collected for a range of different purposes, but the processing for CARRIER involves the processing of newly gathered data as well as the processing of already gathered data for the prevention and treatment of ASCVD. This means that the processing relies on the manner in which the data have been gathered in the first place and how the different applicable legal regimes impact upon that processing.

CARRIER is a project primarily taking place in the Netherlands. Therefore, it is subject to the European Union General Data Protection Regulation (GDPR), which has direct effect in European Union Member States’ law and the national law implementing the GDPR. The implementing law enables the Netherlands to make choices in relation to discretions contained in the GDPR. Alongside the personal data protection laws, the law relating to medical devices must be taken into consideration, as CARRIER seeks to create a digital health solution. SN is a partner in the project and holds one of the data sets that is key to the development of the project. The rules under which personal data held by SN can be accessed or processed by third party partners is strictly governed under the SN act. Finally, besides legal considerations, there are ethical considerations, particularly in relation to ‘nudging’ people to adopt particular behaviors (ie, using an eHealth solution to adopt a healthy lifestyle), and more broadly, about the ethics of individuality and solidarity in relation to the use of personal data for developing novel CPMs for ASCVD.

Federated learning, as is explained previously, enables communication of pseudonymized data from different organizations. Federated learning might therefore provide safeguards and techniques that enable the sharing of nonidentifiable answers to questions asked of the data between the stakeholders, thereby contributing to public health while respecting individuals’ privacy. However, how this federated data infrastructure can be organized and accepted within the requirements of law and ethics is one of the goals of CARRIER.

In order to make this assessment, CARRIER follows 3 broad phases. First, it ensures that the research undertaken—particularly in relation to the development of CPMs using already gathered data—conforms to the law as it is currently understood. Second, it identifies alternative interpretations sustainable in the law to ensure the future operation of the digital health solution and the continuing research. this part acknowledges that GDPR (and local implementing law) presents a number of options for, for example, processing data in the public interest. Further, the operation of informed consent is far from clear in GDPR. Given that there are legitimate options available for the interpretation of GDPR, CARRIER is exploring how those interpretations of the law can work in relation to the work of the project. One aspect of this exploration is to present to different publics (eg, patients, citizens, and policymakers) the dilemma that citizens want both new and effective treatments that are dependent upon big data processing techniques, and at the same time, they want control of their privacy. This public engagement produces a set of answers that feeds into the last phase. Last, it engages with regulatory authorities, whereby CARRIER will present to the European Data Protection Board and the Dutch Supervisory Authority the findings of the second phase of the work. The aim is to produce a dialogue around the interpretation of GDPR that is open to different understandings of the central dilemma (as explained above), and the interpretation of autonomy and solidarity in the use of personal data in (big data) research.

## Conclusion

Digital health is a promising tool for the prevention and treatment of ASCVD. In recent years, many digital health solutions have been developed, but barriers, as described by the Society of Cardiology e-Cardiology working group, exist for a successful large-scale implementation. In this paper, we presented solutions as proposed by the CARRIER consortium.

We described the development process of a digital health solution, employing design thinking and cocreation with relevant stakeholders. Using cocreation, we ensure that the digital health solution meets the needs and vision of future end users. Personalization of the eHealth solution can improve adherence to the intervention. This enhances the eHealth implementation in care pathways for ASCVD.

We also described the modeling approach for a screening model to identify people at high risk for ASCVD. CPMs that can make use of conventional and nonconventional risk factors from different organization create the opportunity for early identification and guiding interventions, even without the need for medical testing.

However, a data infrastructure connecting these different organizations is currently not available. We described the possibilities and characteristics of a federated data infrastructure that enables the connection of these organizations. The Personal Health Train allows for federated data analysis while keeping data owners in control of their data. As this federated data infrastructure raises ethical and legal questions, we also described the development of a framework that ensures responsible data handling in health care.

We believe the future of health care is data driven and supported by eHealth. Therefore, our research on a mature and sustainable federated data infrastructure and ethical and legal aspects will not only facilitate CARRIER but may also facilitate other future health care initiatives.

## Abbreviations

ASCVD: atherosclerotic cardiovascular disease
CARRIER: Coronary ARtery disease: Risk estimations and Interventions for prevention and EaRly detection
CPM: clinical prediction model
EHR: electronic health record
ESC: Society of Cardiology
GDPR: General Data Protection Regulation
SMC: secure multiparty computation
SN: Statistics Netherlands
TRIPOD: Transparent Reporting of a Multivariable Prediction Model for Individual Prognosis or Diagnosis
